# The Multifunction of CLAVATA2 in Plant Development and Immunity

**DOI:** 10.3389/fpls.2016.01573

**Published:** 2016-10-24

**Authors:** Lixia Pan, Shuo Lv, Nan Yang, Yanting Lv, Zhijun Liu, Jinbin Wu, Guodong Wang

**Affiliations:** ^1^Key Laboratory of Ministry of Education for Medicinal Plant Resource and Natural Pharmaceutical Chemistry, National Engineering Laboratory for Resource Developing of Endangered Chinese Crude Drugs in Northwest of China, College of Life Sciences, Shaanxi Normal UniversityXi’an, China; ^2^Laboratory of Phytopathology, Wageningen UniversityWageningen, Netherlands

**Keywords:** CLV2, receptor-like kinase, plant development, plant immunity, *Arabidopsis*

## Abstract

The *CLAVATA2* (*CLV2*) gene encodes a leucine-rich repeat receptor-like protein, a class of cell surface receptors that lacks a cytoplasmic kinase domain. As such, CLV2 is capable of functioning in concert with additional receptor(s), possibly receptor-like kinase(s), to activate cellular responses upon ligand perception. Accumulating data indicate that CLV2 is implicated in distinct biological processes including plant growth and development as well as innate immunity to microbe and nematode infections. This article focuses on recent advances in our understanding of multiple signaling pathways mediated by multifunctional CLV2 that modulate various physiological processes. The challenges and future perspectives of elucidating the specificity of CLV2-mediated signaling pathways and identifying potential co-receptors and putative ligands for CLV2 are also discussed.

The *Arabidopsis CLAVATA2* (*CLV2*) gene encodes a leucine-rich repeat (LRR) receptor-like protein (RLP) that lacks a cytoplasmic kinase domain, unlike receptor-like kinase (RLK) such as CLV1 which contains a cytoplasmic kinase domain (Clark et al., 1997; Jeong et al., 1999). It has been shown that, similar to CLV1 and CLV3, CLV2 is implicated primarily in the proper coordination between proliferation and differentiation of stem cells in the shoot apical meristem (SAM) (Clark et al., 1993, 1995, 1997; Kayes and Clark, 1998; Wang et al., 2008). However, unlike *CLV1* and *CLV3, CLV2* is expressed broadly not only in the SAM but also in many other tissues and is induced by multiple external stimuli, which suggests a wider role for *CLV2* beyond SAM maintenance (Kayes and Clark, 1998; Wang et al., 2008, 2010b; Wu J. et al., 2016). Indeed, recent studies have indicated that CLV2 is involved in distinct physiological programs, including plant development and innate immunity to microbe and nematode infections (Wang et al., 2010a; Replogle et al., 2011; Hanemian et al., 2016). Identification of multiple functions of CLV2 raises the question of how the specificities of CLV2-mediated signaling are achieved. Other interesting aspects include the potential crosstalk of CLV2-mediated signaling pathways and how the formation of CLV2-associated receptor complex(es) is controlled. This article presents recent advances in our understanding of multiple functions of CLV2 in various biological processes.

## CLV2 is Implicated in the Regulation of SAM Maintenance

**FIGURE 1:**
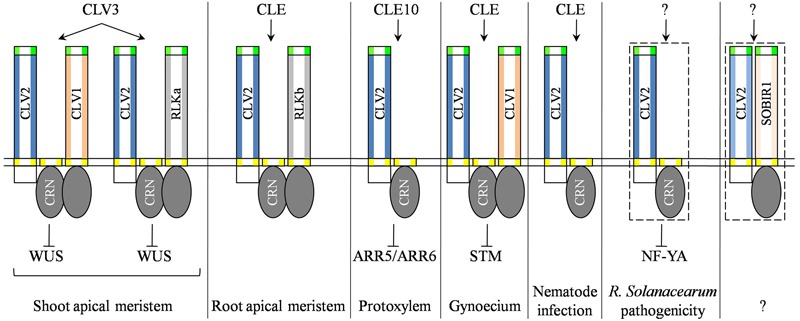
**Schematic representation of different CLV2-associated receptor complexes that mediate CLE signaling in multiple biological processes.** Particularly, the genetic and biochemical evidence are unknown for the physical interaction between CLV2 and CRN, both of which are potentially involved in *R. solanacearum* pathogenicity, and CLV2 associates with SOBIR1 that controls a yet uncharacterized physiological process (dash-line boxed). RLKa and RLKb represent unknown receptors that function together with CLV2 and CRN/SOL2 to form multimers and perceive CLE signals.

Mutations in the *CLV2* gene, similar to *CLV1* and *CLV3*, resulted in enlarged meristems and abnormal organs, while the *clv2* mutant exhibited weaker phenotypes than those of the *clv1* and *clv3* mutants (Clark et al., 1993, 1995, 1997; Kayes and Clark, 1998; Diévart et al., 2003; Wang et al., 2008). CLV3, one of the best studied CLAVATA3/EMBRYO SURROUNDING REGION-related (CLE) peptides (Wang et al., 2016), functions as an intercellular signaling molecule to repress expression of the stem cell-promoting gene *WUSCHEL* (*WUS*), thereby restricting the stem cell population (Fletcher et al., 1999; Brand et al., 2000; Schoof et al., 2000; Rojo et al., 2002; Lenhard and Laux, 2003). The CLV3 peptide is thought to be recognized in parallel by multiple receptor complexes (**Figure 1**), including CLV1 homomultimers, RECEPTOR-LIKE PROTEIN KINASE2/TOADSTOOL2 (RPK2/TOAD2) homodimers, heteromultimers of CLV2 and CORYNE/SUPPRESSOR OF OVEREXPRESSION OF LLP1-2 (CRN/SOL2), and heteromultimers of CLV1 with its close homologs BARELY ANY MERISTEM1 (BAM1) and BAM2 (Casamitjana-Martinez et al., 2003; Miwa et al., 2008; Müller et al., 2008; Bleckmann et al., 2010; Kinoshita et al., 2010; Zhu et al., 2010; Shinohara and Matsubayashi, 2015). Possibly, the CLV3 signal is also perceived by heteromultimers of CLV1 associated with CLV2-CRN/SOL2 (**Figure 1**), based on the result that CLV1 was found to weakly interact with the CLV2-CRN/SOL2 heterodimer (Bleckmann et al., 2010; Zhu et al., 2010). By applying the multiparameter fluorescence imaging spectroscopy (MFIS) method, it is further revealed that CLV homodimers, CLV2-CRN heteromultimers and CLV2-CRN-CLV1 multimers are organized in preformed complexes in the absence of CLV3. The addition of CLV3 stimulates the accumulation of CLV2-CRN-CLV1 multimers within specific domains along the plasma membrane (Somssich et al., 2015).

Specifically, CRN encodes a membrane-associated receptor-like cytoplasmic kinase (RLCK) that lacks a distinct extracellular domain (Miwa et al., 2008; Müller et al., 2008). It has demonstrated that CRN and CLV2 interact at the endoplasmic reticulum and relocalize to the plasma membrane (Bleckmann et al., 2010). Despite CRN kinase being catalytically inactive, the kinase domain of CRN is essential for CLV3 signaling perception in the SAM (Nimchuk et al., 2011; Somssich et al., 2016). Nevertheless, the CLV2-SOL2/CRN heterodimer likely functions together with an unknown RLK, independently of CLV1, to mediate CLV3 signal transduction in the SAM homeostasis (**Figure 1**).

Direct binding of CLV3 peptide with the extracellular LRR domains of CLV1 and BAM1 receptors has been reported (Ogawa et al., 2008; Shinohara and Matsubayashi, 2015). However, CLV2 did not exhibit direct binding to CLV3 peptide, albeit its ability to bind a number of CLE peptides (Guo et al., 2010; Shinohara and Matsubayashi, 2015). On one hand, these results suggest the need for identification of CLV2 ligands, and on the other the results further underscore the hypothesis that an unknown RLK controls SAM homeostasis together with the CLV2-CRN/SOL2 heterodimer (**Figure 1**).

## CLV2 is Implicated in the Regulation of Root Apical Meristem (RAM) maintenance

A growing body of evidence suggests an important role for *CLV2* in root meristem maintenance. Overexpression of many *CLE* genes, including *CLV3, CLE14, CLE19, CLE20*, and *CLE40*, results in an inhibition of root growth in a CLV2-dependent but CLV1-independent manner (Fiers et al., 2004, 2005; Strabala et al., 2006; Meng and Feldman, 2010). Consistently, the *clv2* mutant is insensitive to chemically synthesized CLE peptides, indicating that CLV2 is required for CLE-induced consumption of the root meristem (Fiers et al., 2005; Ito et al., 2006; Kondo et al., 2006; Kinoshita et al., 2007). Similarly, the roots of *crn/sol2* mutants are unaffected upon CLE peptide treatment, suggesting that CRN is also involved in transmitting CLE signals to regulate RAM homeostasis (Miwa et al., 2008; Müller et al., 2008). However, no visible phenotypic abnormalities were observed in roots of the *clv2* mutant, the *crn/sol2* mutant and the *clv2 crn/sol2* double mutant under normal growth conditions (Wang et al., 2008; Miwa et al., 2008; Müller et al., 2008). Possibly, a closely related *AtRLP* gene could compensate for the loss of *CLV2* function in the root (Wang et al., 2010b). It is also likely that the phenotype is very subtle and only become apparent at the microscopic level.

As has been shown in the SAM, CLV2 is also capable of forming a receptor complex with CRN/SOL2 to regulate RAM homoeostasis (**Figure 1**; Miwa et al., 2008; Müller et al., 2008; Bleckmann et al., 2010; Zhu et al., 2010). In addition to the previous observation that CRN lacked kinase activity (Nimchuk et al., 2011), it was reported recently that the CRN/SOL2 kinase domain was not essential for protein function in the root (Somssich et al., 2016). This finding indicates that an unknown receptor, probably a RLK, functions together with the CLV2-CRN heterodimer (**Figure 1**). As such, the root expressed *RLKs*, including *BAMs* and *RPK2*, might be the logical RLK candidates for the redundant role in root development, because of their pronounced expression in roots (DeYoung et al., 2006; Kinoshita et al., 2010). However, it has been shown that BAM1, independently of CLV2, functioned synergistically with RPK2 in CLE peptide-triggered root meristem arrest (Shimizu et al., 2015). An investigation of homozygous T-DNA insertion lines of root expressed *RLKs* for their sensitivity to CLV3 peptide treatment revealed none of the tested RLKs were involved in CLV3 perception (ten Hove et al., 2011). Thus it will be worthwhile in future to examine the homozygous lines with a wide range of CLE peptides (Qiang et al., 2013). A number of *CLE* genes are expressed in the RAM (Jun et al., 2010); however, it is still unclear which CLE peptide is responsible for RAM activity due to the lack of loss-of-function mutants. Nevertheless, it is suggested that CLV2-CRN/SOL2 may perceive the CLE14 and CLE20 ligands to trigger RAM termination, although biochemical evidence for this receptor-ligand interaction is missing (Meng and Feldman, 2010). Altogether, these studies argue that CLV2-CRN/SOL2 heteromers may act together with an unidentified RLK to modulate root meristem maintenance.

## CLV2 is Implicated in the Regulation of Protoxylem Formation

It has been reported that ectopic protoxylem vessels were formed in *clv2* roots (Kondo et al., 2011), suggesting that endogenous CLV2 functions in the inhibition of protoxylem vessel formation. Many chemically synthesized CLE peptides, including CLE10, suppresses protoxylem vessel formation in the root (Kondo et al., 2011). Consistently, transgenic plants overexpressing *CLE10* under an estradiol-inducible promoter exhibited a protoxylem vessel defect similar to that of seedlings treated with CLE10 peptides (Kondo et al., 2011). By contrast, further investigation found that the protoxylem vessel defect is absent in CLE peptides treated *clv2* mutants and *crn/sol2* mutants (Kondo et al., 2011), indicating that CLV2 and CRN/SOL2 may function as receptors, presumably constituting a receptor complex as has been shown in the SAM and RAM, to perceive CLE10 signaling and modulate protoxylem vessel formation (**Figure 1**). Indeed, both *CLV2* and *CRN/SOL2* are strongly expressed in the root stele with greatly overlapping expression domains (Müller et al., 2008; Somssich et al., 2016). However, it remains unclear whether the kinase domain of CRN/SOL2 is involved in the transmission of CLE10 signaling to suppress protoxylem vessel formation. It is most likely that the kinase domain of CRN/SOL2 is dispensable for CRN/SOL2 function in the inhibition of protoxylem vessel formation similar to that in the RAM (Somssich et al., 2016). If this hypothesis holds true, it raises again the possibility that the interaction of CLV2-CRN/SOL2 with an unidentified RLK to form a functional receptor complex (**Figure 1**).

## The *CLV2* Mutant Affects Organ Development

Notably, the inactivation of *CLV2* results in pleiotropic effects on *Arabidopsis* development in addition to enlarged meristem and increased organ number (Kayes and Clark, 1998; Wang et al., 2008, 2011), suggesting a multifactorial role for *CLV2* that is verified by its broad expression pattern (Wang et al., 2008; Wang, 2009; Wu J. et al., 2016). Indeed, mutations in the *CLV2* gene result in phenotypic alterations in the gynoecia, flower pedicels and stamens (Kayes and Clark, 1998). Particularly, it was found that the *clv1, clv2*, and *crn* mutants produced only extra fruit organs and generated floral meristems of similar dimensions to wild-type plants (Durbak and Tax, 2011). Characterization of gynoecium development in these mutants revealed increased cell proliferation and ectopic fruit organ initiation, which was marked by an expanded expression of cell proliferation-promoting gene *SHOOTMERISTEMLESS* (*STM*) (Durbak and Tax, 2011). In contrast to SAM development, in which CLV1 and CLV2-CRN2/SOL2 act in parallel, CLV1, CLV2 and CRN/SOL2 function together in a linear pathway during fruit development based on genetic analyses (Durbak and Tax, 2011).

The *clv2* mutant developed reduced plant size with smaller and narrowed rosette leaves, which is consistent with the observation that *CLV2* exhibited a constitutive expression in rosette leaves (Wang, 2009). Interestingly, overexpression of several *CLE* genes caused phenotypic abnormalities in leaf shape and size (Strabala et al., 2006). Furthermore, *BAM1, BAM2*, and *BAM3* are also implicated in the leaf formation, as the loss of three *BAM* genes caused reduced leaf size (DeYoung et al., 2006). Moreover, *PLL1, PLL4*, and *PLL5*, three downstream genes involving in the CLV signaling pathway of the SAM, have also been shown to be functioned in leaf development (Song and Clark, 2005). Taken together, a CLV-like signaling pathway that includes CLE, CLV2, BAMs, and PLLs probably exists in the regulation of leaf development.

## CLV2 Functions in Plant-Microbe Interactions

Beyond its roles in plant development, CLV2 is also implicated in plant-pathogen interactions. Overexpression of *CLE*-like genes from nematodes resulted in meristem termination and short roots, which mimic the overexpression phenotypes of plant CLE peptides (Replogle et al., 2011). Further studies have shown that CLV2, in conjunction with CLV1, RPK2, and CRN/SOL2, is required for perception of nematode CLE peptides allowing nematodes to successfully infect *Arabidopsis* roots (Replogle et al., 2011). Likewise, it has been shown that CLV1, CLV2, and CRN/SOL2 are required for full susceptibility to virulent strains of *Ralstonia solanacearum* (Hanemian et al., 2016). Interestingly, mutations in *CLV1* and *CLV2* provide resistance not only to *R. solanacearum* strains but also to the oomycete pathogen *Hyaloperonospora arabidopsidis*, suggesting that CLV1 and CLV2 may generally be involved in various plant-pathogen interactions (Hanemian et al., 2016). However, other CLV-signaling components involved in SAM stem cell homeostasis, e.g., CLV3, BAMs, WUS, POL, PLL1, and KAPP, are dispensable for plant susceptibility to *R. solanacearum*, indicating that the signaling pathways employed in *R. solanacearum* pathogenicity differ from those used in stem cell homoeostasis (Hanemian et al., 2016). As such, it is possible that CLV1, CLV2, and CRN/SOL2 control developmental programs that also provide plants to adapt to external stimuli through resource reallocation to balance the growth-immunity tradeoff. Notably, the *clv2* mutant exhibited reduced growth (Wang et al., 2008), which makes biological sense because defense activation generally comes at the expense of plant growth.

The increased disease resistance mediated by *clv1* and *clv2* is independent of salicylic acid and ethylene (Hanemian et al., 2016). Gene expression analysis found that the nuclear transcription factor *Y* subunit alpha (*NF*-*YA*) genes were down-regulated in *clv1* and *clv2* mutants (Hanemian et al., 2016). Consistently, the accumulation of miR169, which is involved in the post-transcriptional regulation of NF-YA transcription factors, is drastically impaired in *clv1* and *clv2* mutants (Hanemian et al., 2016). Similar to the results reported in nematodes, it is speculated that *R. solanacearum*-derived peptides, either CLE-like peptides or unknown peptides, are recognized by CLV1 and CLV2, thereby promoting pathogenicity through the manipulation of intrinsic developmental CLV signaling. Alternatively, it is likely that an unidentified *Arabidopsis* CLE peptide, constituting a signaling pathway possibly with CLV1 and CLV2, may require for *R. solanacearum* pathogenicity.

## Conclusions and Future Perspectives

**FIGURE 2:**
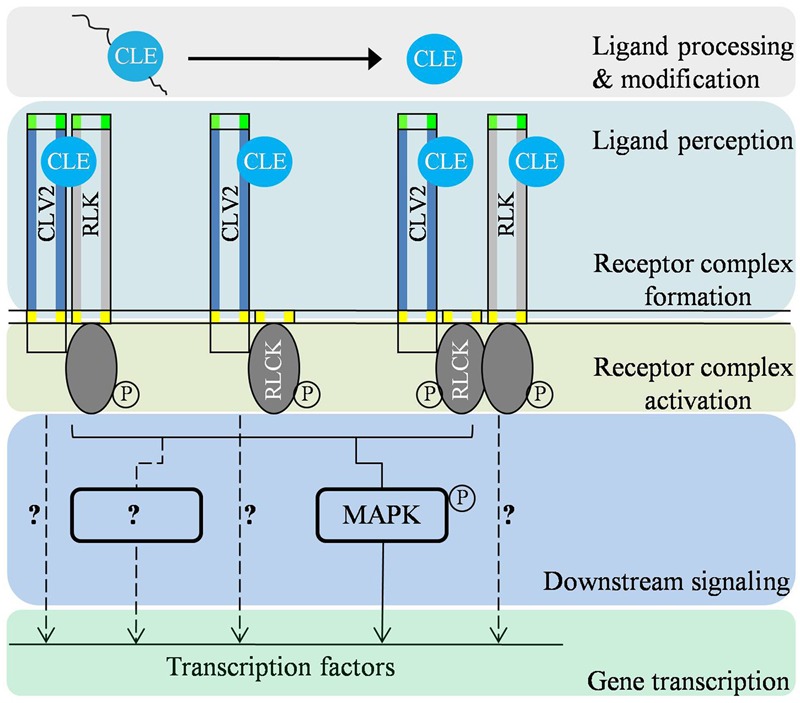
**Predicted signal transduction cascades of CLV2-associated receptor complexes in modulating different biological processes.** Firstly, CLE is proteolytically processed and/or post-translationally modified to be a functional CLE signaling peptide. On the plasma membrane, CLV2 forms receptor complexes with RLK(s)/RLCK(s) and perceive the putative ligand signal. In the cytoplasm, the ligand signal is modulated through regulation of phosphorylation status of the receptor complex. Upon activation, CLV2-associated receptor complexes subsequently transmit the signal to downstream signaling components such as mitogen-activated protein (MAP) kinases and possibly other yet-unidentified components (?). There also may be other mechanisms mediating the signaling output of CLV2-associated complexes (dotted lines with?).

Accumulating data indicate that CLV2 is capable of regulating various developmental and immunity signaling pathways (**Figure 2**). It is well-known that CLV2 needs to interact with additional component(s), possibly with RLK(s), and RLCK(s), to activate cellular responses upon ligand perception because of lacking the intracellular signaling domains (**Figure 2**). The association of CLV2 with different regulatory RLKs and RLCKs might result in activation of distinct biological responses, implying the diversity of CLV2-associated receptor complexes partially determine the specificity of CLV2-mediated signaling. It is also conceivable that ligand-dependent differential phosphorylations of regulatory RLKs and RLCKs could initiate a characteristic response (**Figure 2**). Nevertheless, potential interacting partners for CLV2 are unknown in most cases (**Figure 1**). Therefore, two available resources, the collection of homozygous T-DNA insertion lines for root expressed *RLKs* and the systematic expression atlas of GUS reporter lines for *LRR-RLKs*, are valuable for functional investigation of possible RLK candidates (ten Hove et al., 2011; Wu Y. et al., 2016). Furthermore, the organization and dynamics of different CLV2-associated receptor complexes could be further investigated using MFIS (Somssich et al., 2015). Notably, it has been shown that tomato SUPPRESSOR OF BIR1-1 (SlSOBIR1) associates with a broad range of tomato RLPs that are involved in either plant development or immunity (Liebrand et al., 2013). Specifically, SlSOBIR1 was found to interact with SlCLV2, the tomato homolog of *Arabidopsis* CLV2 (Liebrand et al., 2013). It is therefore hypothesized that, in Arabidopsis, SOBIR1 may also interact with CLV2. The speculation is in line with the fact that *SOBIR* is expressed in many tissues and organs overlapping with *CLV2* (Wu Y. et al., 2016). However, the biological consequence of the interaction between SOBIR1 and CLV2 remains unclear.

Multiple studies have shown the physical interactions between RLKs and G-protein components. For instance, *Arabidopsis* Gβ protein AGB1 was found to interacts with ZAR1 to regulate cell division (Yu et al., 2016), and with ERECTA to control silique development and in response to pathogens (Lease et al., 2001; Llorente et al., 2005). Recently, the heterotrimeric G proteins composed of XLG2/3, AGB1, and AGG1/2 was found to interact with the FLS2-BIK1 complex and regulate immune signaling (Liang et al., 2016). Intriguingly, emerging evidence also exemplifies the interaction between CLV2-like proteins and G-protein components. FEA2, the maize ortholog of CLV2, is found to associate with Gα protein CT2 in coordinating maize SAM maintenance (Bommert et al., 2013). It leaves a question on how the FEA2-CT2 complex transmits the signal intracellularly. However, AGB1 was found to physically interact with RPK2, but not CLV2, to mediate CLV3 signaling (Ishida et al., 2014). The CLV2-dependent and CLV2-indpendent regulation of G-protein signaling potentially provide biochemical plasticity to diverse regulation of stem cell homeostasis. It remains elusive whether any canonical downstream regulators of G-protein signaling or any yet-unidentified components are involved in these processes (**Figure 2**). Altogether, there is little insight on how or whether CLV2 and CLV2-like proteins modulate the G-protein signaling.

Another aspect of future research will be to elucidate the putative ligand(s) for CLV2, which will be helpful in understanding the molecular mechanisms and the specificities of CLV2-mediated multiple biological processes. Thus far, no CLE peptide has been demonstrated as the ligand for CLV2, although it could potentially bind a variety of related CLE peptides (Guo et al., 2010; Shinohara and Matsubayashi, 2015). Inevitably, this raises again the question on how the specificity of CLV2-mediated signaling pathways is achieved to control plant development and innate immunity. Elucidating the potential ligands for CLV2 will also help to clarify the similarity and diversity of the signaling pathways mediated by CLV2.

## Author Contributions

GW, JW, and LP conceived and wrote the manuscript; GW, LP, and SL contributed the figures; NY, YL, and ZL critically reviewed the manuscript.

## Conflict of Interest Statement

The authors declare that the research was conducted in the absence of any commercial or financial relationships that could be construed as a potential conflict of interest.
